# Comparison of Ultrastructural Cytotoxic Effects of Carbon and Carbon/Iron Particulates on Human Monocyte-Derived Macrophages

**DOI:** 10.1289/ehp.7389

**Published:** 2004-11-22

**Authors:** John F. Long, W. James Waldman, Robert Kristovich, Marshall Williams, Deborah Knight, Prabir K. Dutta

**Affiliations:** ^1^Department of Veterinary Biosciences,; ^2^Department of Pathology,; ^3^Department of Chemistry, and; ^4^Department of Molecular Virology, Immunology, and Medical Genetics, Ohio State University, Columbus, Ohio, USA

**Keywords:** carbon, carbon/iron, cytotoxicity, macrophages, ultrastructure

## Abstract

In this study, we tested the hypothesis that the presence of iron in carbon particulates enhances ultrastructural perturbation in human monocyte-derived macrophages (MDMs) after phagocytosis. We used 1-μm synthetic carbon-based particulates, designed to simulate environmental particulates of mass median aerodynamic diameter ≤ 2.5 μm (PM_2.5_). Cultures of human MDMs or T-lymphocytes (as a nonphagocytic control) were exposed to carbon or carbon/iron particulates for various time periods and examined by transmission electron microscopy for ultrastructural changes. T-cells failed to internalize either of the particulates and showed no organelle or nuclear changes. Conversely, MDMs avidly phagocytized the particulates. MDMs treated with C particulates exhibited morphologic evidence of macrophage activation but no evidence of lysis of organelles. In contrast, MDMs treated with C/Fe particulates exhibited coalescence of particulate-containing lysosomes. This phenomenon was not observed in the case of C particulates. By 24 hr there was a tendency of the C/Fe particulates to agglomerate into loose or compact clusters. Surrounding the compact C/Fe agglomerates was a uniform zone of nearly total organelle lysis. The lytic changes diminished in proportion to the distance from the agglomerate. In such cells, the nucleus showed loss of chromatin. Although C particles induced no detectable oxidative burst on treated MDMs, C/Fe particles induced a nearly 5-fold increase in the extracellular oxidative burst by treated MDMs compared with untreated controls. Iron bound to C particles catalyzed the decomposition of hydrogen peroxide to generate hydroxyl radicals. Results of these studies suggest that, among particulates of similar size, biologic activity can vary profoundly as a function of particulate physicochemical properties.

Respirable particulates containing transition metals such as iron, vanadium, nickel, and copper are known to catalyze the generation of reactive oxygen species (ROSs) such as the highly damaging hydroxyl radical (Smith et al. 2000). Sequelae from ROS production can include lipid peroxidation of cell membranes, oxidative modification of proteins, oxidation of DNA, and alterations in calcium homeostasis of the cell.

The process of phagocytosis allows uncontrolled entry of iron-containing particulates into cells because these particulates have bypassed control by the protein transferrin (Hardy and Aust 1995) and its cognate receptor. Transferrin is a transport factor normally found in serum (Darling and Morgan 1994). Although iron is essential for life, its uncontrolled entry into cells has the potential for damaging the cell by oxidative stress (Smith et al. 2000). Iron release from phagocytized iron is greatest at a low pH (e.g., present in lysosomes containing iron particulates), indicating that iron can be mobilized inside macrophages after phagocytosis (Gilmour et al. 1996).

Valavanidis et al. (2000), studying the generation of hydroxyl radicals by urban suspended particulate matter (PM), concluded that the presence of trace metals, especially iron in the form of soluble ferrous and ferric salts, play an important role for oxidant-generating activity. There is also variation in bioactivity of iron in different compounds. Fubini et al. (1999) points out that not every iron-containing solid is active; iron oxide, for example, was mostly inactive.

Our group has examined two naturally occurring zeolites, including forms whose biologic activity is reported to range from highly pathogenic (erionite) to essentially benign (mordenite). We found that on exposure to the same mass of a specific type of particulate, the oxidative burst increases with decreasing particle size but remains relatively independent of zeolite composition. On the other hand, the Fenton reaction depends on the type of zeolite, suggesting that the surface structure of the iron on the zeolite plays an important role (Fach et al. 2002). In another study, we found that the mutagenic potential of mordenite was not enhanced by the addition of ferrous ion. Conversely, mutagenicity of erionite was significantly enhanced by the addition of ferrous ions. These results suggested that although the cytotoxicity of mordenite and erionite may be related to the ability of these fibers to transport iron into a cell, the different coordination state of iron associated with the two fiber surfaces is critical for inducing genotoxic damage (Fach et al. 2003).

In the present study, we used synthetic 1-μm carbon and carbon/iron particulates, designed to simulate environmental particulates of mass median aerodynamic diameter ≤ 2.5 μm (PM_2.5_) that occur as airborne pollution. We made ultrastructural observations and compared human blood monocyte-derived macrophages (MDMs; known to avidly phagocytize PM) with human T-cells (as nonphagocytic controls) on exposure to C or C/Fe particulates. To assess the bioactivity of C and C/Fe particulates, luminescent assays using luminol for oxidative bursts were conducted after exposure of macrophages to the particulates. In addition, the ability of the particulates to produce hydroxyl radicals from hydrogen peroxide (H_2_O_2_) was studied (Fenton reaction).

## Materials and Methods

### Synthesis of C and C/Fe particulates.

The procedures followed were adapted from a previously published procedure for the synthesis of a carbonaceous negative image of a zeolite (Johnson et al. 1997). The synthesis of C particle replicas proceeds through an acid-catalyzed condensation reaction of phenol and paraformaldehyde monomers using zeolite Y as a template for the reaction. The zeolite was acidified by ion exchange with NH_4_Cl, followed by decomposition of the ion-exchanged ammonium ions under vacuum at 500°C. Iron was added to the appropriate particles by ion exchange with 0.01 M ferrous sulfate (3 cycles, 30 min each) before decomposition of the ammonium ions. The zeolite was cooled to room temperature, and solid phenol (0.21 g phenol/g zeolite) was added. A weak vacuum was pulled on the system, and the temperature was raised slowly to 60°C. Solid paraformaldehyde (0.25 g paraformaldehyde/g zeolite) was added to the zeolite/phenol material, and the temperature was raised very slowly to 120°C in a nitrogen environment. At this point the solid material turns red as the phenol/paraformaldehyde cross-linked polymer forms. The solid material was held at 120°C for 5 hr to allow for complete polymerization. The zeolite/polymer mix was pyrolyzed at 800°C for 19 hr under flowing argon. The zeolite template was removed by etching in 48% hydrofluoric acid for 6 hr. The size of the particles was confirmed by scanning electron microscopy (SEM). Bulk analysis of C/Fe particulates found aluminum content of 1.38%, silicon 0.33%, iron 0.46%, and the rest carbon.

### Preparation of target cells.

Peripheral blood mononuclear cells (PBMCs) were separated by Ficoll-Hypaque density gradient centrifugation from fresh heparinized blood collected by venipuncture from healthy, nontransfused consenting volunteers, as previously described (Boyum 1968; Waldman et al. 1992). This study was approved by the Ohio State University institutional review board, human subjects protocol 94HO344. For isolation and differentiation of monocytes, PBMCs were suspended in mononuclear leukocyte culture medium [complete Dulbecco’s modified Eagle’s medium (DMEM)] (Waldman et al. 1992) supplemented with 10 ng/mL monocyte-colony–stimulating factor (M-CSF; R&D Systems, Minneapolis, MN), transferred to six-well plastic tissue culture plates (Costar, Corning Inc., Corning, NY) at a concentration of approximately 2 × 10^7^ cells/well (2 mL/well), and incubated at 37°C in a humidified atmosphere of 10% CO_2_/90% air for 48 hr to allow adherence of monocytes. Nonadherent cells were then removed, and adherent monocytes were washed twice with Seligman’s buffered saline solution (SBSS) and supplied with fresh complete M-CSF–free leukocyte culture medium. Cells were incubated for an additional 10–12 days with medium changes at 48-hr intervals to allow differentiation into the macrophage phenotype and were labeled MDMs.

T-lymphocytes were isolated from PBMCs by negative selection with a commercially available cocktail of monoclonal antibodies and complement (T Lympho-kwik, One Lambda, Inc., Los Angeles, CA), using methods based on those of Clouse et al. (1987) as detailed elsewhere (Waldman et al. 1992). To remove any residual monocytes, cells were incubated in leukocyte culture medium for 1 hr in plastic tissue culture flasks (Costar) at 37°C in a humidified atmosphere of 10% CO_2_/90% air, after which nonadherent cells were recovered for use in experiments.

To assess homogeneity of populations prepared in this manner, samples were suspended in SBSS, stained with fluorescein isothio-cyanate–conjugated monoclonal antibodies specific for CD3, CD4, CD8, and CD14 (Gen Trak, Liberty, NC) as previously described (Waldman et al. 1992), and analyzed (5,000 cells) using a Coulter Epics XL flow cytometer (Beckman Coulter, Inc., Fullerton, CA). As controls for nonspecific staining, cells were reacted with appropriate isotypically matched irrelevant murine antibodies. MDMs routinely marked 90–95% positive for CD14 with undetectable levels of T-cell contamination (as indicated by the absence of CD3^+^ cells). T-cells routinely marked 90–95% positive for CD3 and 25–35% positive for CD8, with the remainder positive for CD4, and had undetectable levels of monocyte contamination (as indicated by the absence of CD14^+^ cells).

### Treatment of cells with particulates.

C/Fe particulates 1 μm in diameter as well as similarly sized C particulates (without iron) were sterilized by steam autoclave and suspended in serum-free DMEM. MDMs differentiated as described above were washed with phosphate-buffered saline (PBS) and supplied with fresh complete DMEM (2.0 mL/well). T-cells were likewise washed and suspended in fresh medium in six-well culture plates (~ 5 × 10^6^ cells in 2 mL/well). Particulates were sonicated and immediately added to cultures at a non-toxic concentration of 5 μg/cm^2^ surface area. Trypan blue dye exclusion was used to demonstrate that the exposure of 5 μg/cm^2^ particles was not toxic to the cells. Plates were centrifuged for 10 min at 300 × *g* immediately after addition of particulates and then incubated at 37°C in a humidified atmosphere of 10% CO_2_/90% air. After various periods of incubation (2–24 hr), MDMs were washed twice with PBS and harvested for fixation by gentle scraping after 15 min of incubation in 0.01% EDTA/PBS at 4°C. Concurrently, free particulates were separated from T-cells by density gradient centrifugation through Ficoll-Hypaque (Histopaque, Sigma, St. Louis, MO), which allows sedimentation of free particulates but not T-cells. T-cells recovered from the gradient were washed twice in PBS before fixation.

### Transmission electron microscopy.

Suspended cells were washed twice in PBS and then fixed for 18–24 hr in 3% cacodylate buffered glutaraldehyde and again pelleted by centrifugation. Cells were washed twice in 0.1 M cacodylate, postfixed in 1% S-collidine–buffered osmium tetroxide (1 hr), and then dehydrated in graded ethanol washes. Specimens were embedded in Medcast (Ted Pella, Inc., Redding, CA). Blocks were cured for a minimum of 12 hr at 60°C. Thin sections (~100 nm) were cut from cured blocks using an ultramicrotome (LKB Nova; LKB, Stockholm, Sweden) and mounted on 2-mm 300-mesh copper grids. Grids were heavy metal stained using a standard two-step uranyl acetate/lead citrate technique and then examined and photographed at 60 kV with a Philips 300 transmission electron microscope (Philips Electronic Instrument Co., Mahwah, NJ).

### Assay of particulate-induced oxidative burst.

Human PBMCs were plated in 96-well Optilux culture plates (BD Falcon, Palo Alto, CA) and allowed to differentiate into MDMs as described above. Immediately before assay, culture medium was removed, and cells were washed twice with PBS before addition of ice-cold serum-free RPMI 1640 (Cellgro-Mediatech, Herndon, VA), 100 μL/well. A stock solution of 100 mM luminol was prepared with 20 mg luminol (5-amino-2,3-dihydro-1,4-phthalazinedione, sodium salt; Sigma)/mL dimethyl sulfoxide and stored at −20°C until use. Immediately before assays, luminol stock solution was thawed, diluted 1:100 (1 mM) in ice cold RPMI, and added to culture wells (100 μL/well). Particulates (C or C/Fe) were sonicated and added to culture wells at a concentration of 5 μg/cm^2^, four replicate wells per treatment. As negative controls, each experiment included 4 replicate wells containing MDMs, medium, and luminol but no particulates. Plates were sealed with UV-sterilized Top Seals (Packard, Meriden, CT) and centrifuged at 4°C (400 × *g*, 3 min). Luminescence was immediately measured (time 0) with a Top-Count Microplate Scintillation and Luminescence Counter (Packard), after which plates were placed in a 37°C incubator. Subsequently, luminescence was measured at 10 min intervals, with plates being incubated at 37°C between counts.

Luminescence indices were calculated by dividing the mean luminescence counts per minute (cpm) of four replicate particulate-treated wells by the mean cpm of four negative control wells at each time point.

### Measurement of hydroxyl radical production by C and C/Fe particles.

Five milligrams of synthetic C or C/Fe particles were weighed and suspended in 500 μL of PBS (pH 7.4) solution. To this suspension, 100 μL of 5,5-dimetheyl-1-pyrolline-*N*-oxide (DMPO; 97% purity; Aldrich Chemical Company, St. Louis, MO) was added, along with 50 μL of a 30% hydrogen peroxide solution (Mallinkrodt Baker, Inc., Phillipsburg, NJ). The solution was shaken in the dark for 15 min. The particles were removed by centrifugation, and the solution was analyzed in a capillary column by electron spin resonance (ESR) spectroscopy (Bruker ESP300 ESR spectrometer; Bruker, Ettlinger, Germany). ESR was performed with a modulating frequency of 100 kHz with a modulating amplitude of 2.090 G. The microwave power was 0.632 mW. The samples were scanned 10 times with the average taken as the representative spectra.

## Results

For the untreated cells (controls), the nucleus showed the expected rim of heterochromatin. The cytoplasmic organelles appeared normal. The microvilli showed expected formations. Nucleoli were ultrastructurally normal.

**Figure 1:**
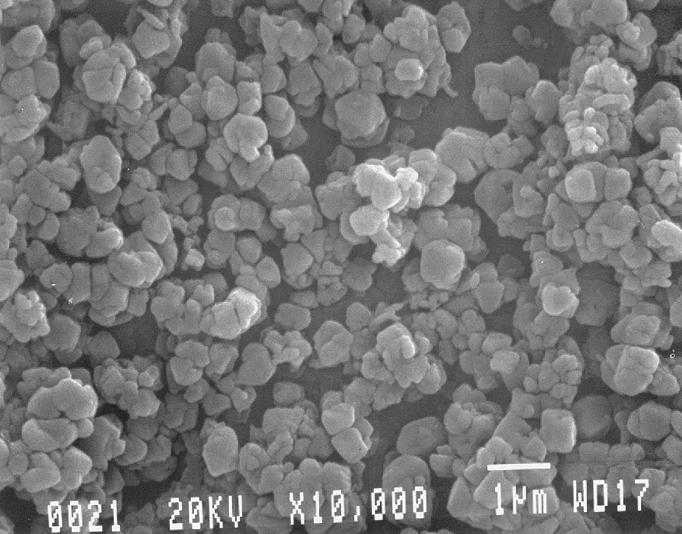
C/Fe particulates seen with SEM (magnification 10,000×). Note size and degree of uniformity. Bar = 1 μm.

An SEM image of the C/Fe synthetic particulates is shown in Figure 1; the uniformity in size is apparent. The C particles are of similar morphology, reflecting the inorganic template used to form both C particles. Because of the extreme heat and acidity required during particle synthesis, there was the expected lack of digestion of the particles in the lysosome. Hence, there was difficulty in morphologically differentiating phagosomes and phagolysosomes in this regard.

### Comparison of C and C/Fe particulates on generating an oxidative burst in the MDMs.

**Figure 2:**
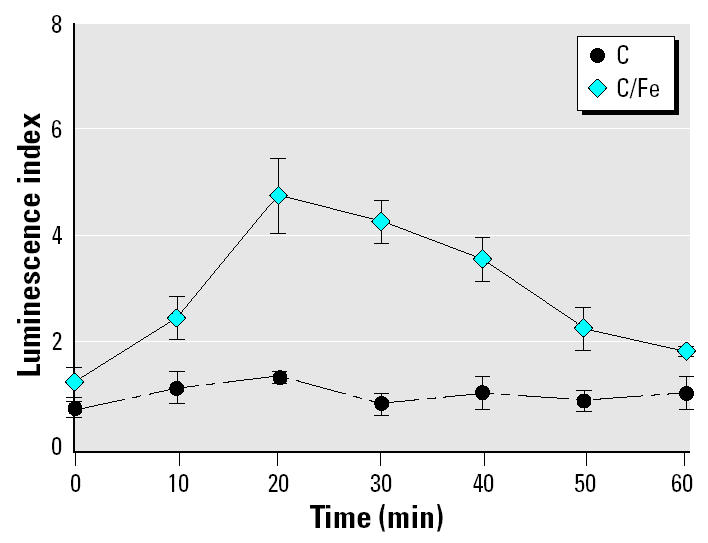
Luminol assay to assess levels of oxidative burst by human MDMs after exposure to C or C/Fe particulates. Error bars indicate mean ± SD of four replicates.

To assess bioactivity related to these synthesized particulates, we performed luminol assays to measure the release of ROSs on phagocytosis (Nadeau et al. 1996). These experiments showed that the oxidative burst reached its peak in the C/Fe-exposed cells after 20 min and then gradually diminished (Figure 2). For the C-exposed cells, no oxidative burst was detected.

Data presented in Figure 2 provide evidence of the differential abilities of the particulates in inducing stress as a function of their physicochemical characteristics. Although C particles induced no detectable oxidative burst in treated MDMs, C/Fe particles induced a nearly 5-fold increase in extracellular oxidative burst by treated MDMs compared with untreated controls.

### Impact of the C particulates on the MDMs.

To examine the kinetics of cell/particulate interaction, we fixed the cells 2–4 and 24 hr after exposure to particulates.

**Figure 3:**
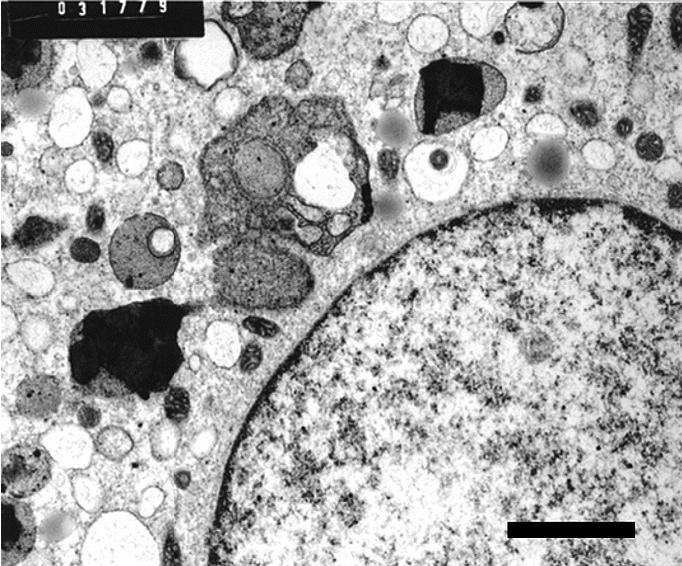
Human MDMs (magnification, 11,000×) 2 hr after exposure to C particulates. Note particulates within intact lysosomes. Bar = 2 μm.

**Figure 4:**
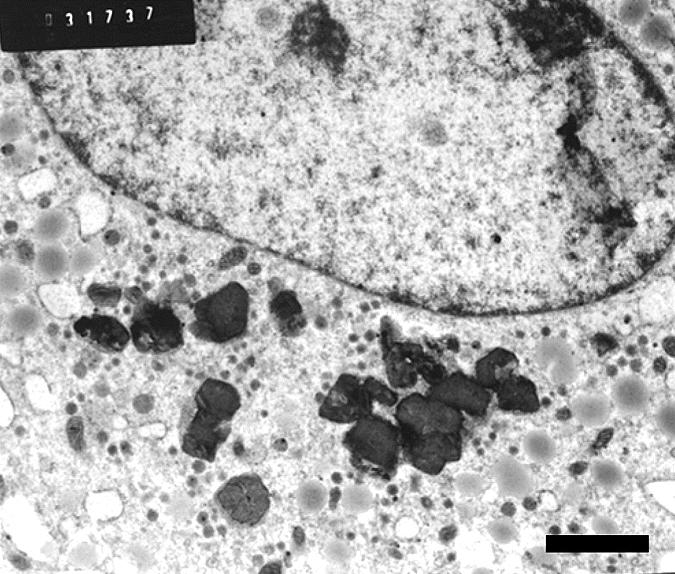
Human MDMs (magnification, 9,000×) 24 hr after exposure to C particulates. Note clumps of individual particulates free in cytoplasm. Bar = 2 μm.

At 2 hr postexposure, ultrastructural signs of macrophage activation were evident with generalized dilatation of endoplasmic reticulum (ER). Numerous C particulates were seen within intact lysosomes (Figure 3). At 24 hr, the C particulates were often in small clumps and without evidence of surrounding lysosomal membrane. There was no evidence of adjacent organelle lysis or of agglomeration of particulates (Figure 4).

### Impact of the C/Fe particulates on the MDMs.

**Figure 5:**
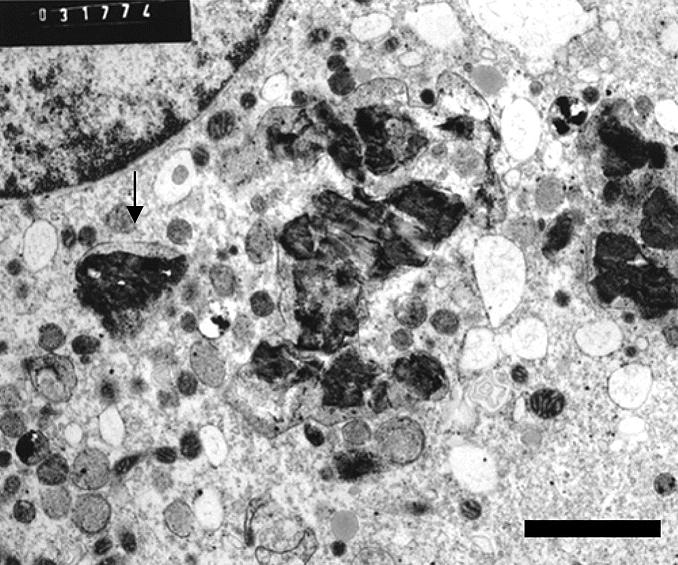
Human MDMs (magnification, 11,000×) at 4 hr after exposure to C/Fe particulates. Note particulates within lysosomes (arrow points to membranes). Note dilatation of ER. Bar = 2 μm.

By the first sampling (4 hr), particulates of similar size and contours as the stock preparation of C/Fe particulates (as seen by SEM) were present within cells. Many particulates were in lysosomes with a detectable membrane surrounding them (Figure 5). In others the membrane could not be followed. Varying degrees of dilation of the ER could be seen in regions adjacent to particulates. The nucleus at this point appeared normal.

**Figure 6:**
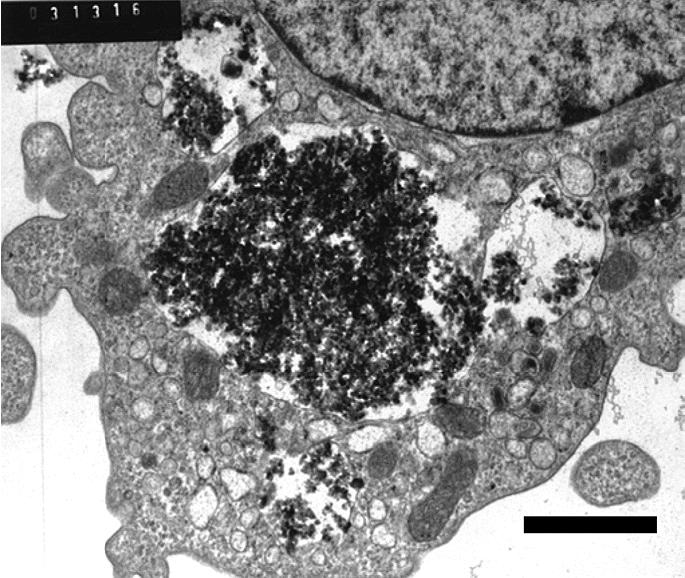
Human MDMs (magnification, 11,000×) 24 hr after exposure to C/Fe particulates. Note tendency for fine particulates to form a loose agglomerate. Some particulates are still within membrane-bound phagolysosomes. The nucleus appears to be nearly structurally normal. Bar = 2 μm.

**Figure 7:**
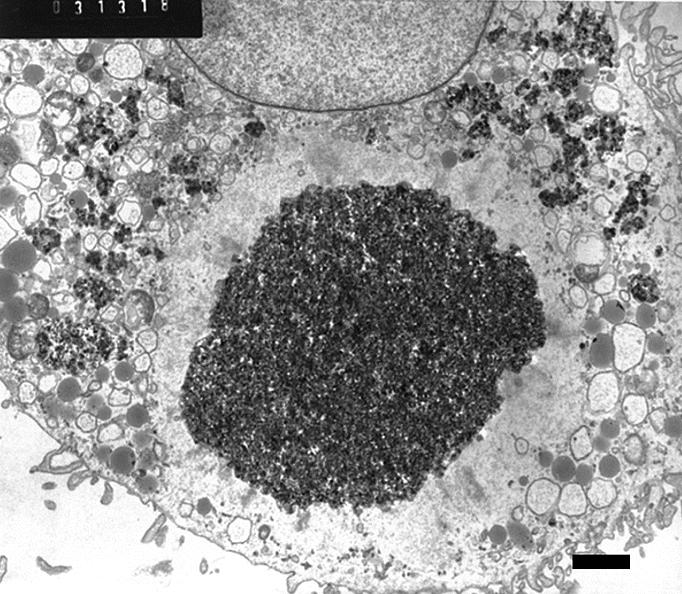
Human MDMs (magnification, 4,900×) 24 hr after exposure to C/Fe particulates. Note chromatolysis of nucleus and the dense cluster of fine particulates with a surrounding zone of lysis. The lysis extends entirely to cell membrane, which is still apparently intact morphologically. Bar = 2 μm.

By 24 hr, the particulates were sometimes partially or markedly aggregated (Figures 6 and 7). Although there was extensive dilatation of the ER, other organelles such as mitochondria appeared morphologically normal. The nucleus at this point continued to appear morphologically normal. In occasional cells (< 10%) still in the 24 hr fixation group, the process seemed more advanced. The agglomerated mass of particulates appeared spherical in overall shape and compact (Figure 7). There was a nearly uniform zone of total organelle lysis surrounding the agglomerate, with less than total lysis surrounding the lytic zone. This lysis extended to include the outer cell membrane. The nucleus by this time had undergone loss of chromatin. The nuclear membrane appeared to still be intact and surrounded the nucleus, which was lacking in chromatin.

### Impact of the C and C/Fe particulates on T-cells.

The control T-cells and T-cells exposed to the C or C/Fe particulates showed no detectable ultrastructural changes. Random fields revealed healthy appearing cells. No evidence of internalized C or C/Fe particulates could be seen in the experimentally exposed group or controls. In both categories, the nuclei were generally oval and had the expected abundant heterochromatin. No ultrastructural changes were detected between the two categories.

### Agglomeration of particulates.

**Figure 8:**
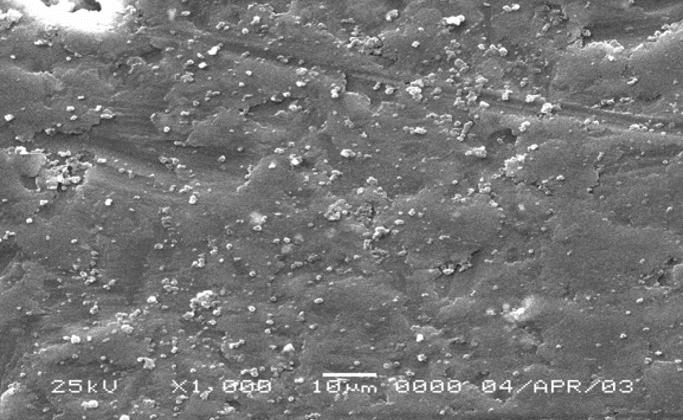
SEM preparation (magnification, 1,000×) after exposure in serum-free medium to suspension of C/Fe particulates. Note no tendency to agglomerate. Bar = 10 μm.

**Figure 9:**
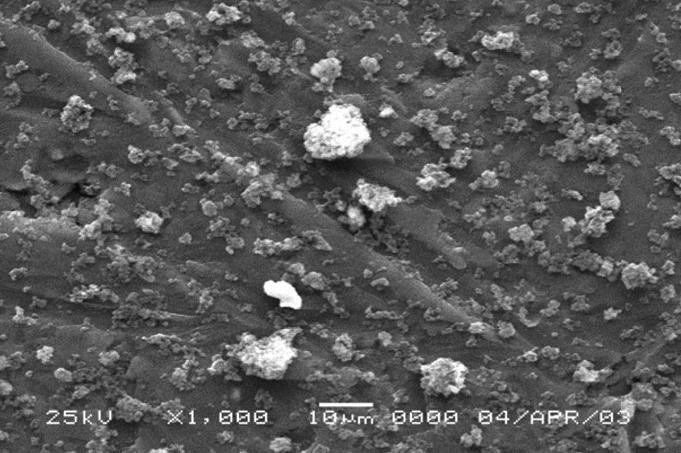
SEM preparation (magnification, 1,000×) after exposure in serum-containing medium to C/Fe particulates. Note tendency to agglomerate in about 10–12 μm clusters. Bar = 10 μm.

To determine the role of serum proteins in the agglomeration of C/Fe particulates, free particulates were incubated for 24 hr in complete or serum-free leukocyte culture medium, washed in distilled water, and examined by SEM. The C/Fe particulates, which had been suspended in the serum-free medium, showed no tendency to agglomerate (Figure 8). In contrast, the C/Fe particulates incubated in complete medium (10% pooled human serum) showed a profound degree of agglomeration (Figure 9) with tightly packed clusters of particulates in a spherical conformation. The clusters were remarkably similar in size (~ 10–12 μm) to those observed intracellularly.

### Particle-bound iron and hydroxyl radical formation.

**Figure 10:**
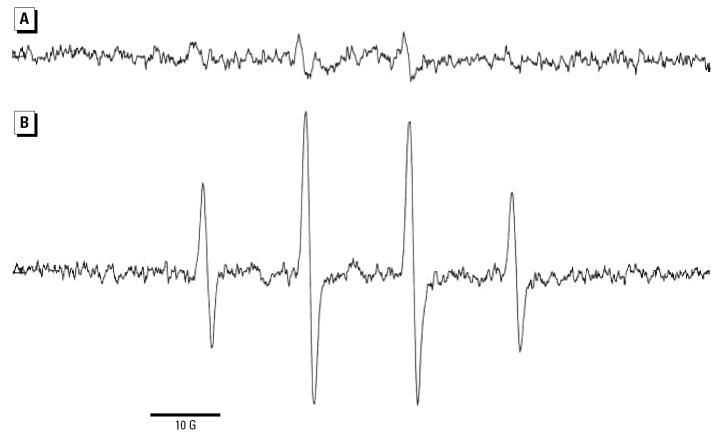
Electronic paramagnetic resonance spectroscopy spectra of the DMPO-OH adduct for C particles (*A*) and C/Fe particles (*B*). Both spectra shown on same scale (10 Gauss).

We observed the propensity of the particles to catalyze the formation of hydroxyl radicals from hydrogen peroxide by ESR spectroscopy. The procedure involved trapping of the hydroxyl radical with DMPO (Shi et al. 2003). The four-line ESR signal (1:2:2:1 quartet) characteristic of 2,2-dimethyl-5-hydroxy-1-pyrrolidinyloxyl (DMPO-OH) with a hyperfine splitting constant of 14.3 G was clearly observed after exposure of C/Fe particles to hydrogen peroxide (Figure 10). The signal after exposure of C particles to hydrogen peroxide and DMPO was smaller by a factor of 10 (Figure 10). Thus, C/Fe particles form hydroxyl radicals with much greater propensity than do C particles. The radicals formed in the presence of carbon must arise from trace levels of iron present in the sample. It is known that the zeolite template itself contains trace iron (McNicol and Pott 1970).

## Discussion

The physical properties of both the C and C/Fe particulates were such that sections for ultrastructural evaluation could be prepared without sectioning artifacts. The occasional absence of lysosomal membranes around particulates (e.g., Figure 4) might be attributed to cell-harvesting procedures. The fact that there was no distortion or displacement of organelles would indicate that the scraping and other aspects of the harvesting procedure did not hinder the interpretation.

Epidemiologic studies have demonstrated that there is a direct correlation between exposure to small airborne particulates and human disease. However, the mechanism(s) by which these particulates exacerbate pulmonary or cardiovascular disease is not known. To begin to address the role of iron in these processes, we synthesized carbon-based particles with or without iron. It was interpreted that cell features associated with activation seen in the MDMs exposed to the C particulates only were a consequence of the activation brought about by the extensive phagocytosis of particulates. There was no lysis of organelles in cells treated with C particles.

Consider the functional significance of some of the C/Fe-exposed cells having intact lysosomal membranes and ER, whereas others did not appear to be related to the length of time of exposure: At the 4-hour sampling, both structures seemed to be morphologically intact (but with dilated ER), whereas at 24 hr, the lysosomal membranes around the agglomerates were often ruptured and there was extensive ER dilation and vacuole formation (approximately in proportion to distance away from the agglomerate). Thus, both time and formation of the large agglomerate were positively correlated with the intracellular lesions. From a functional standpoint, it seems likely that sequelae from generation of ROSs could require time as well as sufficient concentration in an agglomerate to reach the threshold to alter the ultrastructural morphology.

We also observed the following regarding the phenomenon of intracytoplasmic agglomeration of C/Fe particulates: At 4 hr postexposure, numerous particulates had become internalized. These were interpreted as being within phagosomes because the membrane could generally be followed around these variably sized particulates. At 24 hr, the particulates were often seen to be forming into much larger clusters but still generally within the lysosomal membranes. At an apparently more advanced phase, the particulates had sometimes formed a dense spherical cluster, surrounded by a zone of nearly total organelle lysis. The cause of such an intracellular lesion is not certain, but it would appear to be compatible with that resulting from reactive oxygen metabolite generation from the C/Fe particles.

To explain the propensity of the C/Fe particulates to form spherical clusters within cells, we tested cell-free medium with and without serum. The agglomerates formed after the addition of C/Fe particulates (but not with C-only particulates) providing that serum was present. The spherical clusters were often approximately 10 μm in overall diameter whether in the cellular or noncellular system. The reason why the individual particulates in the intracellular clusters were smaller than particulates initially ingested by the macrophage is not apparent. One explanation may be that the large C/Fe conglomerate formation underwent a fracturing process associated with the cutting effect by the microtome blade. Given that during synthesis of the C/Fe particulates, the material is subjected to extreme acidification procedures, the acidification reached in a lysosome would not be sufficient to decompose the particle. Hence, it seems unlikely that degradation on a purely chemical basis could have occurred in the biologic environment of the lysosome and that the apparent smaller particle size is an artifact of the preparation associated with the slicing of a dense agglomeration of graphitic particles.

Regarding the occurrence of agglomerates of particulates observed within cells, two possibilities seem to exist as to their formation. One possibility is that the agglomerates developed outside of the cell in the culture medium (Figure 9) and were subsequently phagocytized as a preformed agglomerate. The demonstrated ability of serum proteins to bring about particulate agglomeration makes this a possibility. It has been recently noted that lung-lining liquid (which would contain surfactant) modifies PM_2.5_ to bring about particle aggregation (Kendall et al. 2002). The other possibility is that individual or small groups of particulates were initially phagocytized and became subsequently agglomerated within the cell. If there were areas of sufficient lysis and loss of intracellular structure in these areas, it would seem feasible that forces (hydrophobicity and others) could enable intracellular clusters of particulates to develop into agglomerates (Figure 7). In our consideration, this last proposal seems more likely in that only individual or small clusters were seen to have been phagocytized in the earliest examinations (4 hr) after exposure of the cell to particulates had occurred.

The T-lymphocytes, similarly exposed, failed to internalize either the C particulates or the C/Fe particulates and subsequently showed no ultrastructural lesions. Thus, we suggest that the failure to internalize the C/Fe particulates avoids the uncontrolled entry of iron into the cell and thus enables the T-lymphocytes to avoid the particulate-induced damage.

Although the oxidative burst of MDMs exposed to the C/Fe particulates occurred early (~20 min) postexposure, the morphologic evidence of ultrastructural change was not detected until much later. The lesions were not seen at the 4 hr sampling but were seen at the 24 hr sampling. The reason the morphologic changes were not evident until later is not clear. One possibility is that cell injury may have occurred early but did not manifest morphologic changes until later. Another possibility is that the lesions were a consequence of the concentration of the possibly toxic products resulting from the coalescence of multiple small phagolysosomes with their contents. Still another possibility is that the physical compression on adjacent organelles as a consequence of the mass of the agglomerate might be considered. Of these, it seems most feasible that the lesions were related to concentration of possibly toxic secondary products produced by a chemical reaction from the agglomerate. The C/Fe particulates contain iron on the surface, and the cell has been demonstrated to undergo an oxidative burst on phagocytosis of the particulates. A likely oxidant produced by the cell during the oxidative burst is hydrogen peroxide. The C/Fe particle can promote the formation of hydroxyl radicals via the Fenton reaction:


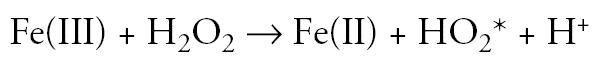



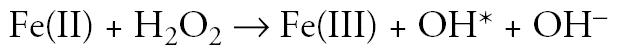


The ultrastructural changes noted in this study are consistent with macrophage activation and damage, which could be explained by the uncontrolled formation of ROSs.

## Summary and Conclusions

Synthetic C and C/Fe particulates (1 μm) were given to cultures of human T-cells and MDMs. The T-cells failed to ingest either particles and showed no ultrastructural changes. The MDMs avidly ingested both type of particles. In contrast, those receiving C particulates showed only ultrastructural changes associated with cell activation. Those receiving C/Fe particulates by 24 hr showed evidence of clustering and coalescence of particulates. A highly discrete, concentrated mass of particulates was sometimes surrounded by a zone of total organelle lysis. Evidence that the C/Fe particulates were bioactive was demonstrated by a nearly 5-fold increase in oxidative burst by treated MDMs. Similar cells exposed to C particulates showed no increase in this regard. The synthetic C/Fe particulates also produced hydroxyl radicals on exposure to hydrogen peroxide. We hypothesize that the formation of intracellular ROSs is responsible for the ultrastructural changes observed. Results of these studies demonstrate that particle-induced ultrastructural changes depend on phagocytosis and suggest that, among respirable particulates of similar size, biologic activity can vary profoundly as a function of particulate physicochemical properties.
